# High prevalence of poly cystic ovary syndrome among young Saudi medical students: an observational cross-sectional study

**DOI:** 10.3389/fmed.2026.1782062

**Published:** 2026-04-20

**Authors:** Thamer Alsufayan, Musaad Alsahly, Hoor K. Aloraini, Thamir Al-khlaiwi

**Affiliations:** 1 Department of Physiology, College of Medicine, King Saud University, Riyadh, Saudi Arabia; 2 College of Medicine, King Saud University, Riyadh, Saudi Arabia

**Keywords:** awareness, medical students, polycystic ovary syndrome (PCOS), prevalence, Saudi Arabia

## Abstract

**Introduction:**

Polycystic ovary syndrome (PCOS) is a common endocrine disorder among women of reproductive age. This study aimed to determine self-reported prevalence of physician-diagnosed PCOS among young medical students at King Saud University and to compare it with reported rates from Western and international populations of similar age. In addition, it assessed the awareness among female medical students at King Saud University.

**Methods:**

A cross-sectional study was conducted using a structured self-administered questionnaire distributed to female medical students at King Saud University between December 2024 and March 2025. The survey contained sociodemographic data, PCOS-related signs and symptoms, past medical history, lifestyle factors, knowledge about PCOS and its complications. Statistical analyses included bivariate analysis and multivariable logistic regression.

**Results:**

We collected 303 responses. The self-reported prevalence of physician-diagnosed PCOS was 18.5%. Common symptoms included hair loss (60.7%), acne (49.8%), and weight gain (32%). PCOS diagnosis was significantly associated with age group (*p* = 0.015), BMI (*p* = 0.038), menstrual irregularities (*p* < 0.001), hirsutism (*p* < 0.001), weight gain (*p* = 0.004), diabetes mellitus (*p* < 0.001), and family history (*p* < 0.001). Multivariable logistic regression identified hirsutism (OR = 4.36, *p* = 0.005) to be significantly associated with self-reported physician-diagnosed PCOS.

**Conclusion:**

The observed prevalence in young medical students in this study was higher than that reported in several Western populations of similar age. Recognition of contributing factors such as genetic susceptibility and lifestyle patterns is essential. Greater emphasis on early screening and targeted health education is recommended.

## Introduction

Polycystic ovary syndrome (PCOS) is one of the most common endocrine disorders affecting women of reproductive age worldwide and is considered a multifaceted hormonal and metabolic condition that influences several organ systems (1). It is classified as a multisystem disorder because it affects adrenal, reproductive, and pituitary hormonal pathways, including abnormalities in adrenocorticotropic hormone (ACTH), gonadotropins, and growth hormone regulation (2).

Clinically, PCOS is characterized by menstrual irregularities, acne, hirsutism, alopecia, anovulation, infertility, and recurrent miscarriages. Hormonally, it presents with elevated androgens, luteinizing hormone, and prolactin. Metabolic features include insulin resistance, obesity, dyslipidemia, and increased risk of impaired glucose tolerance progressing to type 2 diabetes mellitus (3). Approximately, worldwide, half of affected women are obese with excess weight which has been shown to exacerbate the condition (4). Additionally, PCOS has been linked to higher rates of anxiety and depression (5).

Global prevalence estimates vary widely, ranging from 4 to 21% depending on the diagnostic criteria and population studied (6). Regional studies further demonstrate this variability; a prevalence of 6.3% was reported in Sri Lanka (7), 9.13% among Indian adolescents (8), and 4.7–4.8% among White women and 3.4–8% among Black women in the United States (9, 10). In Saudi Arabia, previous research reported a self-reported prevalence of approximately 16% (11). These estimates generally include women across the entire reproductive age span. PCOS prevalence may vary across different age groups within the reproductive period (2, 6). Globally, PCOS prevalence and burden have increased over the past decades, with 65.77 million cases in 2021 and higher rates in middle Socio-Demographic Index regions, especially among women aged 45–49 (12). Exploring PCOS prevalence among younger women specific groups—especially medical students—is essential, as they represent future healthcare providers who will diagnose and manage this condition. In addition, evaluating their knowledge and health-seeking behaviors may also reflect broader cultural and regional influences (13, 14).

Therefore, this study aimed to determine the self-reported prevalence of physician-diagnosed PCOS among young medical students at King Saud University, assess their awareness of PCOS and its complications, and compare these findings with prevalence reported in Western and global populations. Additionally, the study assessed potential associations between PCOS diagnosis and lifestyle factors, BMI, and family history, which may provide context for understanding regional patterns in prevalence.

## Methods

### Study design

This observational cross-sectional study was conducted in the Department of Physiology, College of Medicine at King Saud University (KSU), Riyadh, Saudi Arabia. Data were collected electronically from female medical students between December 2024 and March 2025 using a structured, close-ended online questionnaire. Using a convenient sampling technique, the study targeted all female medical students enrolled at medical college of KSU during the data collection period who voluntarily agreed to participate. Inclusion criteria: female medical students at King Saud University. Exclusion criteria: non-medical female students and those who did not consent to participate. The online questionnaire was distributed via social media platforms such as WhatsApp, Instagram, X platforms, and university email. It was developed in English and consisted of five sections: 1. Sociodemographic characteristics (age, medical year, weight, height, social status). 2. PCOS-related signs and symptoms. 3. Past medical history. 4. Lifestyle factors. 5. Knowledge about PCOS and its complications. PCOS diagnosis was self-reported by participants based on a prior physician or gynecologist diagnosis. Students who reported a diagnosis were asked to confirm that it had been established during follow-up at King Khalid University Hospital, where the Rotterdam criteria are routinely applied in clinical practice. The investigators did not independently verify diagnoses, and no direct clinical assessment was performed as part of this study. In addition, the questionnaire assessed factors that may be associated with PCOS diagnosis, including BMI, menstrual irregularities, hirsutism, family history, and lifestyle habits, allowing comparison of prevalence with previously published international data. Ethical approval was obtained from the Institutional Review Board (IRB) of King Saud University, Riyadh, Saudi Arabia (reference: E-24-9389). The minimum required sample size was calculated using the single-proportion formula:


$$n=\frac{\;Z^{2}P(1-P)}{d^{2.}}$$

where Z = 1.96 (95% confidence level), d = 0.05, and P = estimated prevalence (19–20%) based on previous studies (15). The calculated sample size was 246 participants. A total of 303 student responses were ultimately collected.

### Statistical analysis

Data were analyzed using IBM SPSS Statistics (Version 27; Armonk, NY, United States). Visualization of results was performed using Microsoft Excel 365. Quantitative variables were summarized using minimum, maximum, mean, and standard deviation. Qualitative variables were presented as frequencies and percentages. Associations between categorical variables were examined using the Pearson chi-square test. Multivariable logistic regression was performed to identify independent predictors of PCOS diagnosis. A *p*-value < 0.05 was considered statistically significant.

## Results

### Sociodemographic characteristics of study participants

A total of 303 female medical students at King Saud University participated in this study. Table 1 shows the sociodemographic characteristics of the 303 medical student participants. The majority of participants were in their first (*n* = 30.0%), or fourth year (*n* = 18.8%) of medical school, and most were between 21 and 23 years old (49.8%). Nearly all participants were single (98.7%). The distribution of BMI categories showed that more than half of participants (57.8%) were within the normal weight range, while a smaller proportion were underweight (15.8%), overweight (16.8%), or obese (7.6%).

**Table 1:** Characteristics of study participants (*N* = 303).

Characteristic	Frequency, n (%)
Medical school year	
First year	91 (30)
Second year	54 (17.8)
Third year	52 (17.2)
Fourth year	57 (18.8)
Fifth year	44 (14.5)
Internship year	5 (1.7)
Age group	
18–20 years old	132 (43.6)
21–23 years old	151 (49.8)
≥ 24	20 (6.6)
Social status	
Divorced	1 (0.3)
Married	3 (1)
Single	299 (98.7)
Body mass index (BMI)	
Underweight (< 18.5)	48 (15.8)
Normal weight (18.5–24.9)	175 (57.8)
Overweight (25.0–29.9)	51 (16.8)
Obese (≥ 30)	23 (7.6)

### Signs and symptoms of PCOS among participants

Table 2 shows the self-reported signs and symptoms of PCOS among the participants. Regarding menstrual cycle length, 84.8% reported a normal cycle (21–35 days), while 13.2% experienced oligomenorrhea (cycles longer than 35 days), and 2.0% reported menorrhagia (heavy or prolonged bleeding). Excessive hair growth on the face or chest (hirsutism) was reported by 22.8% of participants, while a larger proportion (60.7%) reported experiencing hair loss (alopecia). Almost half of the participants reported having acne (49.8%), and 19.8% reported skin discoloration (acanthosis nigricans) in the neck or underarm areas. Weight increase was reported by 32.0% of the sample.

**Table 2:** Prevalence of polycystic ovary syndrome signs and symptoms.

Signs and symptoms	Answer	Frequency	Percentage
How long is the usual duration of time between the start of your 2 periods?	Normal (21–35 days)	257	84.8%
	Menorrhagia (Heavy or prolonged menstrual bleeding)	6	2.0%
	Oligomenorrhea (Longer than 35 days between periods)	40	13.2%
Do you experience excessive hair growth on your face or chest?	No	234	77.2%
	Yes	69	22.8%
Do you complain of an increase in your weight?	No	206	68.0%
	Yes	97	32.0%
Do you have hair loss?	No	119	39.3%
	Yes	184	60.7%
Do you have acne?	No	152	50.2%
	Yes	151	49.8%
Do you have black discoloration in your neck or underarm?	No	243	80.2%
	Yes	60	19.8%
If you are married, do you have difficulty getting pregnant?	No	6	2.0%
	Not married	294	97.0%
	Yes	3	1.0%
If you are married, did you undergo any medical intervention to get pregnant?	No	8	2.6%
	Not married	293	96.7%
	Yes	2	0.7%
Have you been diagnosed with Diabetes Mellitus?	No	291	96.0%
	Prediabetic	9	3.0%
	Yes	3	1.0%
Do you take any medications for diabetes?	No	152	50.2%
	Not diabetic	144	47.5%
	Yes	7	2.3%
Do you have any women in your family with PCOS symptoms or the symptoms mentioned above?	No	175	57.8%
	Yes	128	42.2%

Regarding diabetes status, 96.0% reported no diagnosis of diabetes mellitus, 3.0% reported being prediabetic, and 1.0% reported having diabetes. Only 2.3% reported using antidiabetic medications. Finally, a family history of PCOS symptoms or similar symptoms was reported by 42.2% of the participants, while 57.8% reported no such family history.

**Figure 1:**
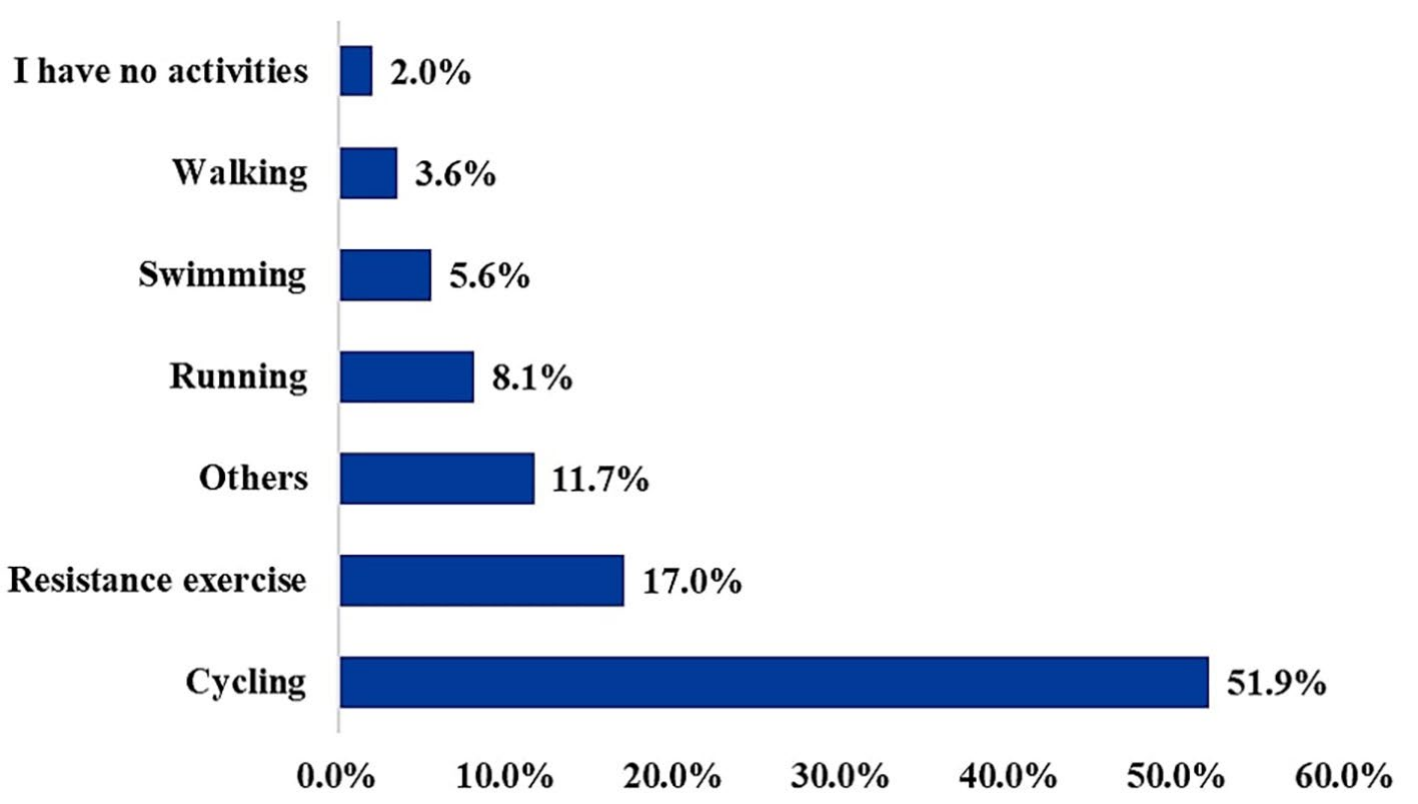
Types of physical activities performed by participants.

Figure 1 displays the proportion of participants who reported undergoing various hormonal tests relevant to PCOS evaluation. A considerable percentage of participants (30.6%) reported that they had not performed any of these tests. Among the tests conducted, thyroid-stimulating hormone (TSH) was the most frequently reported (12.4%), followed by follicle-stimulating hormone (FSH) at 11.2%, and luteinizing hormone (LH) at 9.5%. Prolactin was tested in 8.2% of participants, estrogen in 6.7%, and testosterone in 6.2%. Tests for plasma insulin, progesterone, androsterone, and androstenedione were reported by smaller percentages of participants (5.5, 4.7, 3.0, and 2.0%, respectively).

**Figure 2:**
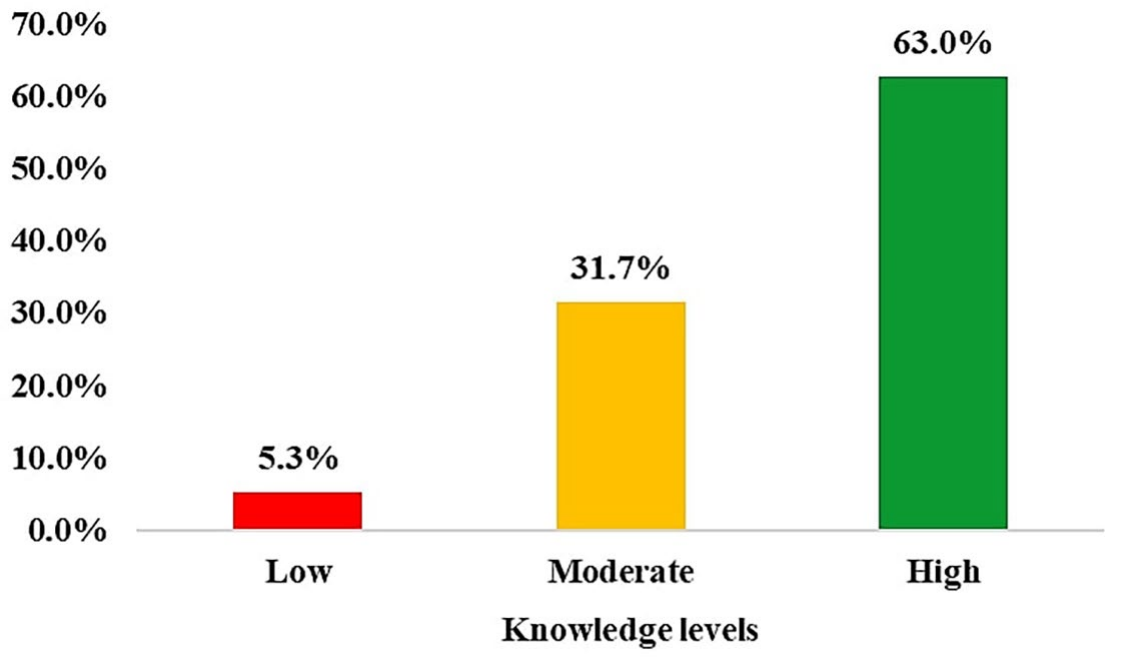
Distribution of knowledge levels regarding PCOS among participants.

Figure 2 illustrates the distribution of abnormal results among the participants who reported having undergone hormonal testing. Among those with abnormal results, prolactin abnormalities were the most frequently reported (5.9%), followed by TSH abnormalities (3.0%). FSH, LH, and plasma insulin abnormalities were each reported by 2.3% of this subgroup. Testosterone abnormalities were slightly less common (1.6%), and estrogen abnormalities were reported by 1.3% of the participants. Androstenedione, Progesterone, and Androsterone abnormalities were the least frequently reported, each at 1.0%.

### Past medical history of participants

Table 3 shows the past medical history related to PCOS among the participants. Regarding ovarian ultrasound examination, 30.0% reported having undergone the procedure, while 70.0% had not. Of those who had an ultrasound, 20.1% had results indicating polycystic ovaries, 14.2% had normal results, and 65.7% of the total sample reported not having done the ultrasound. Concerning glucose testing, 26.4% of participants reported having undergone an oral glucose tolerance test or fasting blood glucose test, while 73.6% had not. Among those who had glucose testing, 25.1% had normal results, 1.7% had abnormal results and most of the sample, 73.3% reported not having had the test. Finally, regarding a prior PCOS diagnosis, 18.5% reported having been diagnosed with PCOS by a doctor or gynecologist, 23.4% reported not having been diagnosed, and a majority (58.1%) reported not having visited a doctor or gynecologist for PCOS evaluation.

**Table 3:** Past medical history related to polycystic ovary syndrome.

Items	Answer	Frequency	Percentage
Did you undergo ovaries examination with ultrasound?	No	212	70.0%
	Yes	91	30.0%
If the answer was yes, did the ultrasound results pointed to polycystic ovaries??	I did not do it	199	65.7%
	No	43	14.2%
	Yes	61	20.1%
Did you undergo oral glucose tolerance test or fasting blood glucose test before?	No	223	73.6%
	Yes	80	26.4%
If the answer was yes, was it normal or abnormal?	Abnormal	5	1.7%
	I did not do it	223	73.3%
	Normal	75	25.1%
Have you been diagnosed with PCOS by any doctor or gynecologist?	I did not go to a doctor or gynecologist for PCOS	176	58.1%
	No	71	23.4%
	Yes	56	18.5%

### Lifestyle of participants

Table 4 shows the lifestyle factors of the participants. Regarding physical activity, 25.1% reported no physical activity, 44.6% reported less than 5 h per week, 23.1% reported 5–10 h per week, and 7.3% reported more than 10 h per week. For fast food consumption, 8.6% reported not eating fast food, 72.6% reported consuming it less than 5 times per week, 18.2% reported 5–10 times per week, and 0.7% reported more than 10 times per week. Concerning soft drink consumption, 31.0% reported not drinking soft drinks, 53.8% reported consuming them less than 5 times per week, 10.6% reported 5–10 times per week, and 4.6% reported more than 10 times per week.

**Table 4:** Lifestyle factors of study participants.

Items	Answer	Frequency	Percentage
What is the duration of your physical activity?	I have no physical activity	76	25.1%
	< 5 h/ week	135	44.6%
	5–10 h/ week	70	23.1%
	> 10 h/ week	22	7.3%
How often do you eat fast food during the week?	I do not eat fast food	26	8.6%
	< 5 times/ week	220	72.6%
	5–10 times/ week	55	18.2%
	> 10 times/ week	2	0.7%
How often do you drink soft drinks during the week?	I do not drink soft drinks	94	31.0%
	< 5 times/ week	163	53.8%
	5–10 times/ week	32	10.6%
	> 10 times/ week	14	4.6%

**Figure 3:**
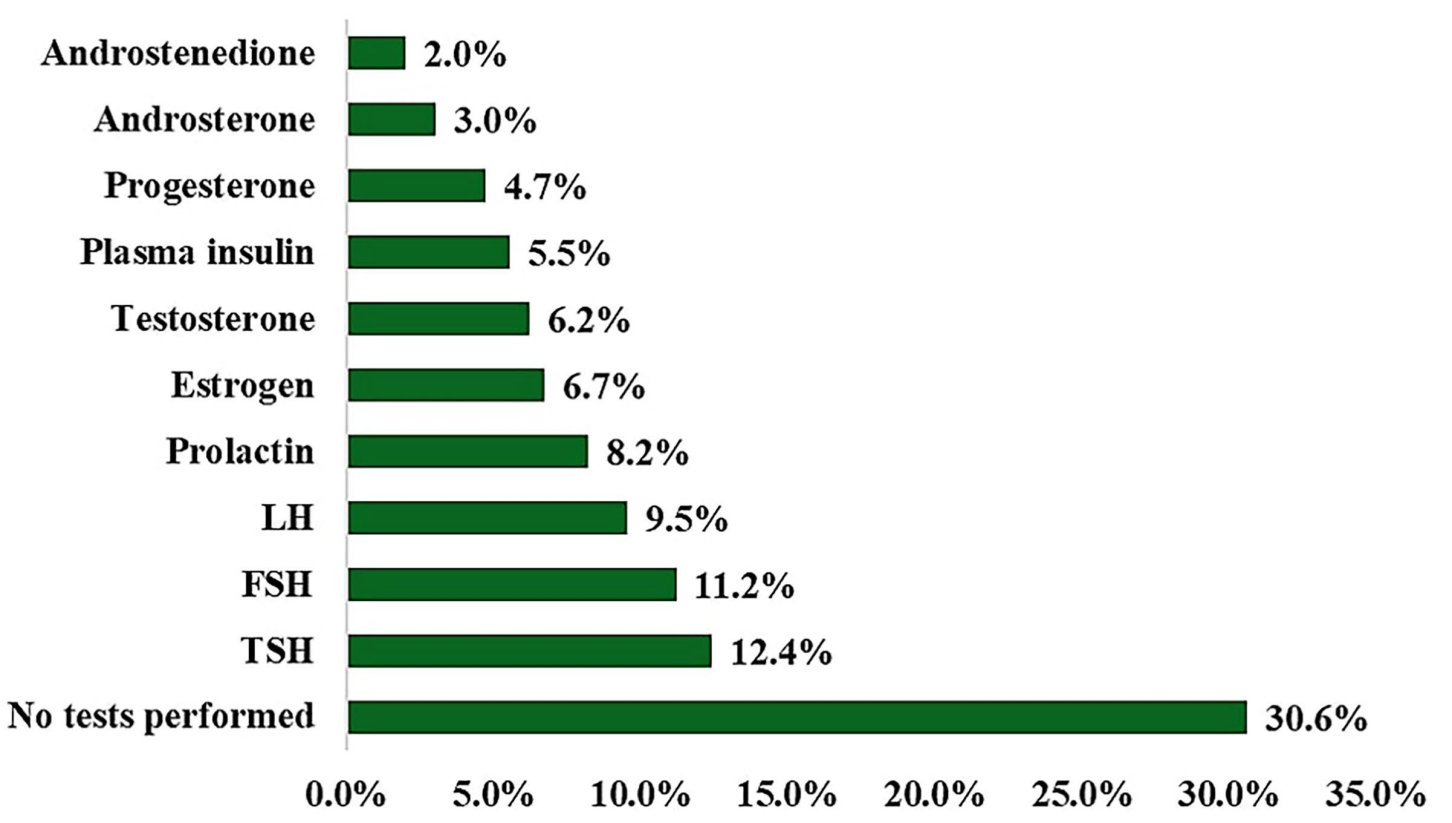
Frequency of hormonal tests performed among participants.

Figure 3 presents the types of physical activity that participants reported engaging in most frequently. Cycling was the most commonly reported activity; 51.9% of participants indicated it as their usual choice. Resistance exercise was the second most frequent, reported by 17.0% of the physically active participants. Other activities were reported by 11.7%, followed by running (8.1%), swimming (5.6%), and walking (3.6%). A small proportion (2.0%) reported having no physical activity.

### Knowledge of PCOS and its complications

Table 5 assesses participants’ knowledge regarding PCOS and its potential complications. A large majority of participants correctly recognized that a healthy diet and regular exercise can help manage PCOS symptoms (93.1%) and that weight loss can decrease PCOS symptoms and signs (87.5%). Most participants were aware that PCOS can lead to diabetes (73.3%) and increase the risk of infertility (94.1%). Knowledge regarding the link between PCOS and cardiovascular disease (heart disease and hypertension) was slightly lower; 64.4% answered correctly. Awareness of the potential association between PCOS and breast/uterine cancer was the lowest; 63.0% answered correctly. The mean total knowledge score (out of a possible 6) was 4.75 ± 1.41, indicating a generally good level of knowledge.

**Table 5:** Participant knowledge of polycystic ovary syndrome and its complications.

Items	No		Yes	
	N	%	N	%
A healthy diet and exercise help manage PCOS	21	6.9%	282	93.1%
Weight loss decreases PCOS symptoms	38	12.5%	265	87.5%
PCOS can lead to Diabetes?	81	26.7%	222	73.3%
PCOS can lead to heart disease/hypertension	108	35.6%	195	64.4%
PCOS can increase infertility risk	18	5.9%	285	94.1%
PCOS can lead to breast/uterine cancer	112	37.0%	191	63.0%
Total knowledge score (of 6)	Mean ± SD		4.75 ± 1.41	

**Figure 4:**
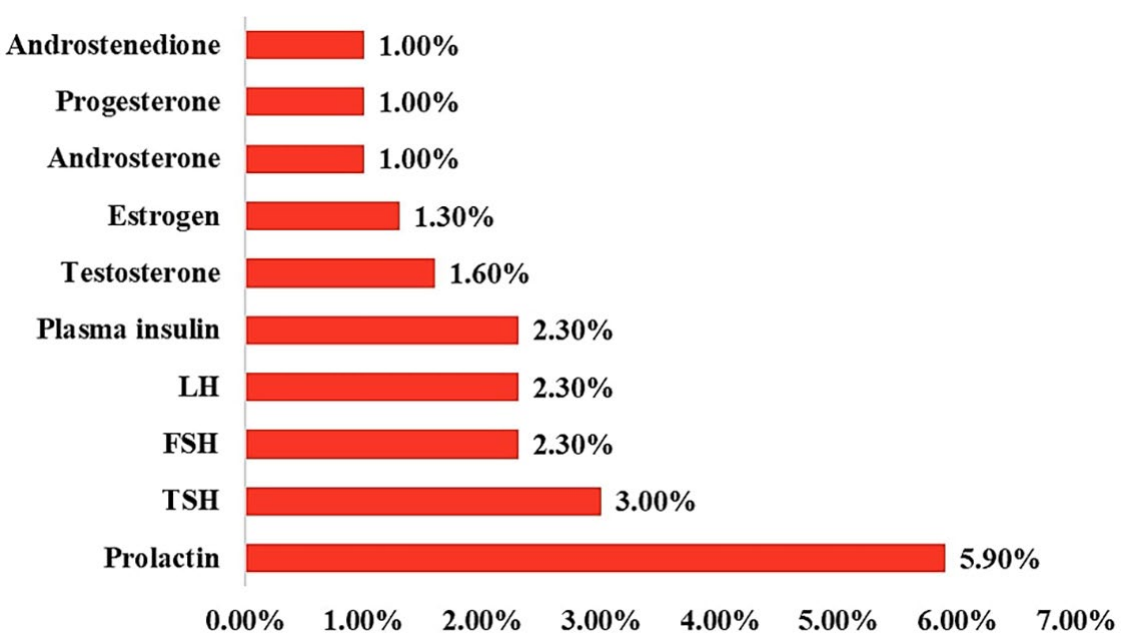
Distribution of abnormal hormonal test results among participants.

Figure 4 demonstrates the distribution of participants across three categorized knowledge levels: low, moderate, and high. The majority of participants (63.0%) were classified as having high knowledge about PCOS and its complications. An extensive proportion (31.7%) demonstrated moderate knowledge. A small percentage of participants (5.3%) were categorized as having low knowledge.

### Association between variables and PCOS diagnosis

Table 6 presents the association between demographic factors and PCOS diagnosis. A statistically significant association was found between age group and PCOS diagnosis (*p* = 0.015). Participants in the 18–20 age group had a lower prevalence of PCOS (13.6%) compared to those in the 21–23 age group (19.9%) and the 24 and above age group (40.0%). BMI was also significantly associated with PCOS diagnosis (*p* = 0.038). Obese participants had a considerably higher prevalence of PCOS (39.1%) compared to overweight (23.5%), normal weight (15.4%), and underweight (16.7%) participants. No significant association was found between the year of medical school and PCOS diagnosis (*p* = 0.290).

**Table 6:** Association between demographic factors and polycystic ovary syndrome diagnosis.

Demographic factor	Answer	Diagnosis with PCOS: No	Diagnosis with PCOS: Yes	*p*-value
Overall PCOS diagnosis		*N* = 247 (81.5%)	*N* = 56 (18.5%)	
Year of Medical School	First Year	78 (85.7%)	13 (14.3%)	0.290
	Second Year	45 (83.3%)	9 (16.7%)	
	Third Year	39 (75.0%)	13 (25.0%)	
	Fourth Year	49 (86.0%)	8 (14.0%)	
	Fifth Year	33 (75.0%)	11 (25.0%)	
	Internship Year	3 (60.0%)	2 (40.0%)	
Age Group	18–20 years old	114 (86.4%)	18 (13.6%)	0.015*
	21–23 years old	121 (80.1%)	30 (19.9%)	
	24 and above	12 (60.0%)	8 (40.0%)	
BMI categories	Underweight	40 (83.3%)	8 (16.7%)	0.038*
	Normal weight	148 (84.6%)	27 (15.4%)	
	Overweight	39 (76.5%)	12 (23.5%)	
	Obese	14 (60.9%)	9 (39.1%)	

The *p*-value is calculated by a Pearson Chi-square test. *significant at *p* ≤ 0.05. Bold values indicate statistically significant results (*p* ≤ 0.05).

Table 7 shows the association between reported signs and symptoms and PCOS diagnosis. Several signs and symptoms were significantly associated with a PCOS diagnosis. Menstrual cycle irregularities were strongly associated with PCOS (*p* < 0.001). Specifically, all participants reporting menorrhagia (100%) and a significant proportion reporting oligomenorrhea (42.5%) had a PCOS diagnosis, compared to only 12.8% of those with a normal cycle. Excessive hair growth (face/chest) was also significantly associated with PCOS (*p* < 0.001); 49.3% of those reporting this symptom had a diagnosis, compared to only 9.4% of those without. Reported weight increase was significantly more common in the diagnosed group (27.8% vs. 14.1%, *p* = 0.004). A significant association was also found between the use of medical intervention to get pregnant and PCOS diagnosis (*p* = 0.010). A diagnosis of diabetes mellitus (*p* < 0.001) and the use of diabetes medication (*p* < 0.001) were strongly associated with PCOS diagnosis. All participants with either condition was in the PCOS-diagnosed group. A family history of PCOS symptoms was also significantly more common among those diagnosed with PCOS (32.8% vs. 8.0%, *p* < 0.001). In contrast, no statistically significant associations were found between PCOS diagnosis and hair loss, acne, skin discoloration, or difficulty getting pregnant, *p* > 0.05.

**Table 7:** Association between polycystic ovary syndrome signs and symptoms and diagnosis.

Signs and symptoms	Answer	Diagnosis with PCOS: No	Diagnosis with PCOS: Yes	*p*-value
Overall PCOS diagnosis		*N* = 247 (81.5%)	*N* = 56 (18.5%)	
Menstrual Cycle Length	Normal	224 (87.2%)	33 (12.8%)	<0.001*
	Menorrhagia	0 (0.0%)	6 (100.0%)	
	Oligomenorrhea	23 (57.5%)	17 (42.5%)	
Excessive Hair Growth (Face/Chest)	No	212 (90.6%)	22 (9.4%)	<0.001*
	Yes	35 (50.7%)	34 (49.3%)	
Weight Increase	No	177 (85.9%)	29 (14.1%)	0.004*
	Yes	70 (72.2%)	27 (27.8%)	
Hair Loss	No	102 (85.7%)	17 (14.3%)	0.130
	Yes	145 (78.8%)	39 (21.2%)	
Acne	No	121 (79.6%)	31 (20.4%)	0.389
	Yes	126 (83.4%)	25 (16.6%)	
Skin Discoloration (Neck/Underarm)	No	202 (83.1%)	41 (16.9%)	0.146
	Yes	45 (75.0%)	15 (25.0%)	
Difficulty Getting Pregnant (If married)	No	5 (83.3%)	1 (16.7%)	0.797
	Not married	240 (81.6%)	54 (18.4%)	
	Yes	2 (66.7%)	1 (33.3%)	
Medical Intervention for Pregnancy (If married)	No	6 (75.0%)	2 (25.0%)	0.010*
	Not married	241 (82.3%)	52 (17.7%)	
	Yes	0 (0.0%)	2 (100.0%)	
Diabetes Mellitus Diagnosis	No	242 (83.2%)	49 (16.8%)	<0.001*
	Prediabetic	5 (55.6%)	4 (44.4%)	
	Yes	0 (0.0%)	3 (100.0%)	
Diabetes Medication Use	No	132 (86.8%)	20 (13.2%)	<0.001*
	Not diabetic	115 (79.9%)	29 (20.1%)	
	Yes	0 (0.0%)	7 (100.0%)	
Family History of PCOS Symptoms	No	161 (92.0%)	14 (8.0%)	<0.001*
	Yes	86 (67.2%)	42 (32.8%)	

The *p*-value is calculated by a Pearson Chi-square test. *significant at *p* ≤ 0.05. Bold values indicate statistically significant results (*p* ≤ 0.05).

Table 8 shows the association between past medical history variables and PCOS diagnosis. A highly significant association was found between ovarian ultrasound examination and PCOS diagnosis (*p* < 0.001). Only 1.4% of participants without a PCOS diagnosis had undergone an ultrasound, compared to 58.2% of those with a diagnosis. Among those who had an ultrasound, the results were also strongly associated with PCOS diagnosis (*p* < 0.001). Of participants with a polycystic ultrasound result, 86.9% had a PCOS diagnosis, whereas 2.3% with a normal ultrasound had a diagnosis. Conversely, there was no statistically significant association between having had an OGTT or fasting blood glucose test and PCOS diagnosis (*p* = 0.080); however, a higher percentage of those diagnosed with PCOS had undergone testing (25.0% vs. 16.1%). The results of the glucose tests (if performed) also showed no statistically significant association with PCOS diagnosis (*p* = 0.092).

**Table 8:** Association between past medical history and polycystic ovary syndrome diagnosis.

Diagnostic Test/Result	Answer	Diagnosis with PCOS: No	Diagnosis with PCOS: Yes	*p*-value
Overall PCOS diagnosis		*N* = 247 (81.5%)	*N* = 56 (18.5%)	
Ovarian Ultrasound Examination	No	209 (98.6%)	3 (1.4%)	<0.001*
	Yes	38 (41.8%)	53 (58.2%)	
Ultrasound Result (If Performed)	I did not do it	197 (99.0%)	2 (1.0%)	<0.001*
	No (Normal)	42 (97.7%)	1 (2.3%)	
	Yes (Polycystic)	8 (13.1%)	53 (86.9%)	
Oral Glucose Tolerance Test (OGTT) or Fasting Blood Glucose Test	No	187 (83.9%)	36 (16.1%)	0.080
	Yes	60 (75.0%)	20 (25.0%)	
OGTT/Fasting Blood Glucose Result (If Performed)	Abnormal	3 (60.0%)	2 (40.0%)	0.092
	I did not do it	187 (84.2%)	35 (15.8%)	
	Normal	57 (75.0%)	19 (25.0%)	

(%) represents the number and percentage within each PCOS diagnosis group. *significance at *p* ≤ 0.05. Bold values indicate statistically significant results (*p* ≤ 0.05).

Table 9 shows the association between lifestyle factors (physical activity, fast food consumption, soft drink consumption), knowledge level, and PCOS diagnosis. No statistically significant associations were found between any of these factors and PCOS diagnosis. The distribution of physical activity duration (*p* = 0.909), fast food consumption (*p* = 0.602), and soft drink consumption (*p* = 0.961) were similar between those with and without a PCOS diagnosis. Similarly, knowledge level (low, moderate, or high) was not significantly associated with PCOS diagnosis (*p* = 0.554).

**Table 9:** Association between lifestyle factors, knowledge level, and polycystic ovary syndrome diagnosis.

Items	Answer	Diagnosis with PCOS: No	Diagnosis with PCOS: Yes	*p*-value
Overall PCOS diagnosis		*N* = 247 (81.5%)	*N* = 56 (18.5%)	
Physical Activity Duration (per week)	< 5 h/week	111 (82.2%)	24 (17.8%)	0.909
	5–10 h/week	55 (78.6%)	15 (21.4%)	
	> 10 h/week	18 (81.8%)	4 (18.2%)	
	I have no physical activity	63 (82.9%)	13 (17.1%)	
Fast Food Consumption (per week)	< 5 times/week	180 (81.8%)	40 (18.2%)	0.602
	5–10 times/week	46 (83.6%)	9 (16.4%)	
	> 10 times/week	1 (50.0%)	1 (50.0%)	
	I do not eat fast food	20 (76.9%)	6 (23.1%)	
Soft Drink Consumption (per week)	< 5 times/week	132 (81.0%)	31 (19.0%)	0.961
	5–10 times/week	27 (84.4%)	5 (15.6%)	
	> 10 times/week	11 (78.6%)	3 (21.4%)	
	I do not drink soft drinks	77 (81.9%)	17 (18.1%)	
Knowledge level	Low	12 (75.0%)	4 (25.0%)	0.554
	Moderate	76 (79.2%)	20 (20.8%)	
	High	159 (83.2%)	32 (16.8%)	

Table 10 presents the results of a multivariable logistic regression model predicting PCOS diagnosis. The model was statistically significant (χ^2^(9) = 176.281, *p* < 0.001). The model explained a real proportion of the variance in PCOS diagnosis, with a Cox & Snell R-Square of 0.448 and a Nagelkerke R-Square of 0.722. The model showed an overall accuracy of 90.9%, correctly classifying 94.2% of those without PCOS and 76.8% of those with PCOS.

**Table 10:** Multivariable logistic regression analysis of factors associated with polycystic ovary syndrome diagnosis.

Predictors	OR (95% CI.)	SE.	Wald	*p*-value
Age group (Ref = 18–20 years old)			1.077	0.584
Age group (21–23 years old)	1.70 (0.601–4.81)	0.531	0.999	0.318
Age group (24 and above)	1.79 (0.214–15.1)	1.086	0.289	0.591
BMI	0.980 (0.488–1.97)	0.356	0.003	0.956
Menstrual Cycle Length (Ref = Normal)	3.09 (0.963–9.95)	0.596	3.600	0.058
Excessive hair growth on face/chest (Ref = No)	4.36 (1.57–12.1)	0.520	7.999	0.005*
Weight Increase (Ref = No)	3.62 (0.991–13.2)	0.661	3.785	0.052
Family history of PCOS/symptoms (Ref = No)	2.74 (0.941–7.99)	0.546	3.419	0.064

OR, Odds Ratio; CI, Confidence Interval; SE, Standard Error. Ref, Reference Category. *significance at *p* ≤ 0.05. Bold values indicate statistically significant results (*p* ≤ 0.05).

After adjusting for other factors, excessive hair growth on the face or chest (hirsutism) was significantly associated with increased odds of PCOS (OR = 4.36, 95% CI: 1.57–12.1, *p* = 0.005). On the contrary, menstrual cycle length (*p* = 0.058), weight increase (*p* = 0.052), and family history of PCOS or its symptoms (*p* = 0.064) approached statistical significance; however, it is still not significant, *p* > 0.05 threshold. Age group and BMI were not significantly associated in the multivariable model.

## Discussion

The present study revealed, through self-reported physician-diagnosed, a prevalence of 18.5% in young Saudi medical students, which is notably higher than rates commonly reported in Western populations for similar age groups, where estimates typically range between 3.4 and 8% (9, 10). Similar prevalence rates have been reported in studies conducted in Middle Eastern and South Asian populations, where prevalence is frequently elevated, often ranging from 16 to 27% (16–18). Such regional variability highlights the importance of considering demographic, genetic, cultural, and lifestyle contexts when interpreting PCOS prevalence across populations. Recent national population-based data from Saudi Arabia also reported a high PCOS prevalence of 22.5% and demonstrated significant associations with sociodemographic and lifestyle factors, further supporting the regional pattern observed in our finding (19).

In line with previous research, several symptoms and factors in our study were significantly associated with PCOS diagnosis. Menstrual irregularities, especially oligomenorrhea, were strongly associated with PCOS (*p* < 0.001), which is consistent with studies identifying menstrual disturbance as one of the most reliable clinical indicators of the disorder (17, 20, 21). Hirsutism was also highly associated with PCOS in the multivariable regression model (aOR = 4.36). This finding aligns with the central role of hyperandrogenism in the pathophysiology and clinical presentation of PCOS, as described in multiple studies (17, 21). Weight gain and obesity were also more common among the PCOS group in the univariable analysis, reflecting the well-established association between adiposity, hyperandrogenism, and insulin resistance (4, 22). Recent findings have noted lipid profile alterations among PCOS patients, aligning with its broader metabolic changes (23).

Family history of PCOS or related symptoms was significantly associated with diagnosis in univariable analysis, consistent with studies demonstrating familial aggregation and a possible genetic predisposition (17, 24). This observation may reflect underlying genetic or environmental differences compared with Western cohorts; however, such interpretations should be made cautiously. While our study did not directly investigate genetic markers, the significant association with family history emphasizes the importance of hereditary influence in PCOS risk.

Interestingly, lifestyle variables—including physical activity, fast-food consumption, and soft-drink intake—showed no significant association with PCOS diagnosis. This finding differs from some international studies that have identified lifestyle as an important modifiable contributor to PCOS severity or progression (22). However, lifestyle influences are complex and may act cumulatively or at the population level rather than being detectable within a single student cohort. Despite the lack of statistical significance in our study, lifestyle patterns common in the region, such as lower physical activity and higher caloric intake, may still contribute to the broader epidemiological differences observed between the Middle East and Western countries.

The participants demonstrated a high overall level of knowledge, with a mean score of 4.75/6 and 63% categorized as having high knowledge. This is consistent with other studies involving medical or health sciences students, who generally possess better understanding of PCOS symptoms and consequences (25–27). However, certain gaps were evident—particularly in knowledge related to long-term complications such as cardiovascular disease and cancer. Similar gaps have been reported in regional studies among Saudi women diagnosed with PCOS (28). This highlights the need for enhanced educational programs that emphasize the chronic nature of PCOS and its systemic health consequences, rather than focusing solely on reproductive aspects.

Overall, the higher prevalence observed in young medical students in our study compared with Western cohorts may reflect a multifactorial interplay between genetic predisposition, higher regional rates of obesity, metabolic risk factors, and cultural or environmental factors. Although lifestyle variables did not reach statistical significance within our cohort, their potential broader population-level impact cannot be dismissed. The findings underscore the importance of early screening, improved awareness of long-term complications, and targeted public health strategies to address PCOS in young women—particularly in regions where prevalence appears elevated.

## Limitations

This study has several limitations. First, its cross-sectional design precludes causal inference. Second, the use of convenience sampling through social media and email may have introduced selection bias and limits generalizability. Third, PCOS diagnosis was self-reported and not independently verified by the investigators, which introduces potential recall and reporting bias. Additionally, the exclusive inclusion of female medical students—who may have higher health literacy and distinct health-seeking behaviors compared with the general population—further limits external validity. Finally, the study was conducted at a single academic center, and findings should not be extrapolated to all young Saudi women.

## Conclusion

This study identified a self-reported PCOS prevalence of 18.5% among young medical students at King Saud University—a rate noticeably higher than the global and Western estimates commonly reported for women of similar age. This observed prevalence within this study population suggests a potentially higher burden compared with some international reports; however, findings should be interpreted within the context of the study population.

These findings underscore the importance of locally derived data when evaluating PCOS prevalence within specific academic populations. Early screening for menstrual irregularities and hyperandrogenic symptoms is therefore essential in this population.

Although awareness among students was generally high, gaps remained regarding long-term metabolic and cardiovascular risks. Strengthening targeted health education and improving access to timely clinical evaluation may help reduce delayed diagnosis and prevent future complications.

## Data Availability

The raw data supporting the conclusions of this article will be made available by the authors, without undue reservation.
